# Circulating HBV RNA and Hepatitis B Core–Related Antigen Trajectories in Persons With HIV/HBV Coinfection and Hepatitis B Surface Antigen Loss During Tenofovir Therapy

**DOI:** 10.1093/infdis/jiae189

**Published:** 2024-04-16

**Authors:** Lorin Begré, Anders Boyd, Marie-Laure Plissonnier, Barbara Testoni, Luisa Salazar-Vizcaya, Franziska Suter-Riniker, Caroline Scholtès, Charles Béguelin, Jürgen K Rockstroh, Huldrych F Günthard, Alexandra Calmy, Matthias Cavassini, Hans H Hirsch, Patrick Schmid, Enos Bernasconi, Massimo Levrero, Gilles Wandeler, Fabien Zoulim, Andri Rauch, the Swiss HIV Cohort Study, I Abela, K Aebi-Popp, A Anagnostopoulos, M Battegay, E Bernasconi, D L Braun, H C Bucher, A Calmy, M Cavassini, A Ciuffi, G Dollenmaier, M Egger, L Elzi, J Fehr, J Fellay, H Furrer, C A Fux, H F Günthard, A Hachfeld, D Haerry, B Hasse, H H Hirsch, M Hoffmann, I Hösli, M Huber, D Jackson-Perry, C R Kahlert, O Keiser, T Klimkait, R D Kouyos, H Kovari, K Kusejko, N Labhardt, K Leuzinger, B Martinez de Tejada, C Marzolini, K J Metzner, N Müller, J Nemeth, D Nicca, J Notter, P Paioni, G Pantaleo, M Perreau, A Rauch, L Salazar-Vizcaya, P Schmid, R Speck, M Stöckle, P Tarr, A Trkola, G Wandeler, M Weisser, S Yerly

**Affiliations:** Department of Infectious Diseases, Inselspital, Bern University Hospital, University of Bern, Switzerland; Graduate School for Health Sciences, University of Bern, Switzerland; Department of Infectious Diseases, Research and Prevention, Public Health Service of Amsterdam, The Netherlands; Stichting hiv monitoring Amsterdam, The Netherlands; Department of Infectious Diseases, Amsterdam UMC location University of Amsterdam, The Netherlands; Department of Infectious Diseases, Amsterdam Institute for Infection and Immunity, The Netherlands; Cancer Research Center of Lyon (CRCL), UMR Inserm U1052 / CNRS 5286, Lyon, France; IHU Lyon, Lyon Hepatology Institute, Lyon, France; Cancer Research Center of Lyon (CRCL), UMR Inserm U1052 / CNRS 5286, Lyon, France; IHU Lyon, Lyon Hepatology Institute, Lyon, France; Department of Infectious Diseases, Inselspital, Bern University Hospital, University of Bern, Switzerland; Institute for Infectious Diseases, University of Bern, Switzerland; Cancer Research Center of Lyon (CRCL), UMR Inserm U1052 / CNRS 5286, Lyon, France; Laboratoire de Virologie, Institut des Agents Infectieux, Hospices Civils de Lyon, France; Department of Infectious Diseases, Inselspital, Bern University Hospital, University of Bern, Switzerland; HIV Clinic, Department of Medicine I, University Hospital Bonn, Germany; Department of Infectious Diseases and Hospital Epidemiology, University Hospital Zurich, Switzerland; Institute of Medical Virology, University of Zurich, Switzerland; Division of Infectious Diseases, HIV Unit, Geneva University Hospitals, University of Geneva, Switzerland; Division of Infectious Diseases, Lausanne University Hospital, University of Lausanne, Switzerland; Transplantation and Clinical Virology, Department of Biomedicine, University of Basel, Switzerland; Division of Infectious Diseases, Infection Prevention and Travel Medicine, Cantonal Hospital St. Gallen, Switzerland; Division of Infectious Diseases, Ente Ospedaliero Cantonale Lugano, University of Geneva and University of Southern Switzerland, Lugano, Switzerland; Cancer Research Center of Lyon (CRCL), UMR Inserm U1052 / CNRS 5286, Lyon, France; IHU Lyon, Lyon Hepatology Institute, Lyon, France; University of Lyon, University Claude Bernard Lyon 1 (UCBL1), Lyon, France; Department of Hepatology, Hospices Civils de Lyon, Lyon, France; Department of Infectious Diseases, Inselspital, Bern University Hospital, University of Bern, Switzerland; Cancer Research Center of Lyon (CRCL), UMR Inserm U1052 / CNRS 5286, Lyon, France; IHU Lyon, Lyon Hepatology Institute, Lyon, France; University of Lyon, University Claude Bernard Lyon 1 (UCBL1), Lyon, France; Department of Hepatology, Hospices Civils de Lyon, Lyon, France; Department of Infectious Diseases, Inselspital, Bern University Hospital, University of Bern, Switzerland

**Keywords:** HBV RNA, hepatitis B core–related antigen, hepatitis B virus, HIV, kinetics

## Abstract

**Background:**

We evaluated long-term trajectories of circulating hepatitis B virus (HBV) RNA and hepatitis B core–related antigen (HBcrAg) in persons with and without hepatitis B surface antigen (HBsAg) loss during tenofovir therapy in the Swiss HIV Cohort Study.

**Methods:**

We included 29 persons with HIV with HBsAg loss and 29 matched persons with HIV without HBsAg loss. We compared HBV RNA and HBcrAg decline and assessed the cumulative proportions with undetectable HBV RNA and HBcrAg levels during tenofovir therapy using Kaplan-Meier estimates.

**Results:**

HBsAg loss occurred after a median of 4 years (IQR, 1–8). All participants with HBsAg loss achieved suppressed HBV DNA and undetectable HBV RNA preceding undetectable quantitative HBsAg levels, whereas 79% achieved negative HBcrAg. In comparison, 79% of participants without HBsAg loss achieved undetectable HBV-RNA and 48% negative HBcrAg. After 2 years of tenofovir therapy, an HBV RNA decline ≥1 log_10_ copies/mL had 100% sensitivity and 36.4% specificity for HBsAg loss, whereas an HBcrAg decline ≥1 log_10_ U/mL had 91.0% sensitivity and 64.5% specificity.

**Conclusions:**

HBV RNA suppression preceded undetectable quantitative HBsAg levels and had high sensitivity but low specificity for HBsAg loss during tenofovir therapy in persons with HIV. HBcrAg remained detectable in approximately 20% of persons with HBsAg loss and 50% of persons without HBsAg loss.

## Teaser

In persons with HIV/hepatitis B virus (HBV) undergoing long-term tenofovir-containing antiretroviral therapy, HBV RNA suppression precedes hepatitis B surface antigen loss. Combining HBV RNA and hepatitis B core–related antigen decline during the first years of tenofovir therapy could improve predictions of hepatitis B surface antigen loss in novel HBV drug trials.

With approximately 300 million people affected, hepatitis B virus (HBV) infection is a major global health problem and a frequent cause of liver cirrhosis, hepatocellular carcinoma, and death [1]. Persons with HIV and HBV are at even increased risk for liver-related complications and death [2]. Current guidelines recommend lifelong treatment with tenofovir disoproxil fumarate or tenofovir alafenamide as part of the antiretroviral therapy (ART) regimen [3, 4]. Hepatitis B surface antigen (HBsAg) loss substantially reduces the frequency of complications but occurs infrequently [5, 6]. However, some studies observed higher rates of HBsAg loss among persons with HIV as compared with persons without HIV [7–9]. In the Swiss HIV Cohort Study (SHCS; www.shcs.ch), HBsAg loss occurred in 16% of 262 persons with HIV/HBV after starting tenofovir-containing ART [8].

Novel serum markers, including hepatitis B core–related antigen (HBcrAg) and circulating HBV RNA, might improve our understanding of HBsAg loss during antiviral therapy. Moreover, previous studies identified HBcrAg and HBV RNA levels as predictors of hepatocellular carcinoma in persons without HIV [10, 11]. HBcrAg is a composite of hepatitis B core antigen, hepatitis B e antigen (HBeAg), and p22 core-related protein, which are precore/core gene products [12]. It is a surrogate of the size of the transcriptionally active pool of intrahepatic covalently closed circular DNA (cccDNA), the molecular reservoir and transcriptional template of HBV [13–15]. Circulating HBV RNA mainly consists of pregenomic RNA, the template for reverse transcription to HBV DNA, and reflects cccDNA transcriptional activity in the hepatocytes during antiviral therapy [16–19]. Thus, these 2 noninvasive biomarkers may predict functional cure of HBV infection (ie, HBsAg loss). However, data are limited on the long-term trajectories of individuals experiencing HBsAg loss during antiviral therapy, especially persons with HIV/HBV [20, 21].

Taking advantage of a cohort of persons with HIV and HBV with serial stored samples available during tenofovir therapy, we compared the long-term trajectories of HBcrAg and circulating HBV RNA levels in persons with and without HBsAg loss. Moreover, we intended to assess diagnostic criteria of these markers between persons with and without HBsAg loss.

## METHODS

### Study Population and Design

Our study was performed within the SHCS, an ongoing nationwide cohort study including >70% of all persons with HIV undergoing ART in Switzerland [22]. All centers’ local ethical committees approved the cohort study, and participants provided written informed consent. We included 29 SHCS participants with chronic HBV infection (defined as 2 positive HBsAg test results at least 6 months apart) who started tenofovir-containing ART and later achieved HBsAg loss. A stored plasma sample within 1 year before the start of tenofovir and a quantitative HBsAg (qHBsAg) <0.05 IU/mL after starting tenofovir was required for inclusion. Participants with a negative HBsAg test result before the start of tenofovir therapy were excluded. The 29 participants with HBsAg loss were matched 1:1 to 29 persons with HIV fulfilling the same inclusion criteria but without HBsAg loss during tenofovir therapy. Matching was based on age (±10 years), sex at birth, lamivudine treatment prior to tenofovir, and CD4^+^ T-cell count category at the start of tenofovir therapy (<200, 200–349, ≥350 cells/mm^3^).

Our primary outcomes were the cumulative proportion of participants with negative HBV DNA, HBV RNA, and HBcrAg levels during tenofovir-containing ART. Our secondary outcomes were the proportions with a decline ≥1 log_10_ in qHBsAg, HBcrAg, and HBV RNA levels 1 and 2 years after starting tenofovir, as well as the sensitivity and specificity of HBcrAg and HBV RNA declines for HBsAg loss.

We defined baseline as the start date of the first tenofovir-containing ART, and follow-up continued to the last available stored sample before death, loss to follow-up, 6 months after cessation of tenofovir, or database closure on 31 December 2020, whichever occurred first. Follow-up continued in case of interruption of tenofovir therapy when participants resumed therapy at any time later on. The time point of HBsAg loss was defined as the first visit with a qHBsAg measurement <0.05 IU/mL. We defined HBV DNA suppression as <20 IU/mL, an undetectable HBV RNA level as <10 copies/mL, and a negative HBcrAg level as ≤3 log_10_ U/mL.

### Laboratory Analyses

We measured HBcrAg, HBV RNA, HBV DNA, and qHBsAg using stored plasma samples at baseline and after 6, 12, 18, and 24 months and yearly thereafter. We retrieved HBeAg status from available data. HBcrAg was quantified by the Lumipulse G HBcrAg assay on the LUMIPULSE G1200 Analyzer (Fujirebio Europe) according to the manufacturer's instructions. We used a cutoff of 3 log_10_ U/mL to determine HBcrAg positivity, as proposed by Kimura et al [12]. As the assay has a linear range from 3 to 7 log_10_ U/mL, samples with HBcrAg >7 log_10_ U/mL were diluted and retested, as described previously [13]. HBV RNA levels were determined with the COBAS HBV RNA automated investigational assay on the COBAS 6800 system (Roche Molecular Diagnostics), which preferentially detects RNA expressed from cccDNA with a lower limit of detection of 3.3 copies/mL and a linear range between 10 and 10^7^ copies/mL, as described previously [19, 23]. We measured HBV DNA using a commercial quantitative nucleic acid test (COBAS HBV on the COBAS 4800 system; Roche Diagnostics) with a lower limit of detection of 4.4 IU/mL and a linear range from 10 to 1 × 10^9^ IU/mL, or we used measurements determined with accredited assays with a lower limit of detection ≤20 IU/mL during routine clinical care. We quantified qHBsAg using a commercial chemiluminescent microparticle immunoassay (ARCHITECT HBsAg; Abbott) with an initial dilution of 1:500, a sensitivity ≤0.05 IU/mL, and an upper limit of detection of 124 925 IU/mL.

### Statistical Analysis

We modeled HBcrAg and HBV RNA levels over time using linear regression. Follow-up time was modeled by restricted cubic splines with 5 knots located at the 5th, 27.5th, 50th, 72.5th and 95th percentiles to allow for nonlinear trajectories of HBcrAg and HBV RNA [24]. We assessed the proportion of participants with undetectable levels of HBV DNA, HBcrAg, and HBV RNA at baseline and 1, 2, 3, 5, and 10 years after starting tenofovir therapy. We determined the cumulative proportion with negative HBV DNA, HBcrAg, and HBV RNA levels using the Kaplan-Meier method and tested differences between participants with and without HBsAg loss using log-rank tests. We calculated time-dependent area under the receiver operating characteristic curve (AUROC), sensitivity, and specificity of a decline ≥1 log_10_ in qHBsAg, HBcrAg, and HBV RNA levels for the prediction of HBsAg loss at 2 and 5 years of follow-up using the R package *timeROC* [25]. When qHBsAg, HBcrAg, and HBV RNA decline was summarized, missing values were linearly interpolated with the closest neighboring values.

We defined statistical significance as a 2-sided *P* < .05. Statistical analyses were performed with Stata/MP (version 16.0; StataCorp) and RStudio (version 2022.7.2.576; RStudio Team) for R (version 4.2.2; R Core Team).

## RESULTS

### Patient Characteristics

The 58 participants were followed for a median of 12 years (IQR, 8–14). HBsAg loss occurred after a median of 4 years (IQR, 1–8; minimum, 0.5; maximum, 14). Prior to starting tenofovir therapy, 48 of 58 (83%) participants were treated with lamivudine-containing ART for a median of 6 years (IQR, 4–8; Table 1). Among the participants with prior lamivudine therapy, 17 of 24 (71%) with HBsAg loss and 13 of 24 (54%) without loss were treated with lamivudine at the time of tenofovir start (*P* = .37). At baseline, 8 of 29 (28%) participants with HBsAg loss and 7 of 29 (24%) without loss had a suppressed HBV DNA load. Baseline HBcrAg and HBV RNA levels were similar in participants with and without HBsAg loss (Table 1). At the start of tenofovir therapy, 13 of 27 (48%) participants with HBsAg loss and 10 of 24 (42%) without loss were HBeAg positive. During follow-up, all participants with HBsAg loss and 28 of 29 (97%) without loss achieved HBV DNA suppression (*P* > .99).

**Table 1.** Baseline Characteristics of Participants With and Without HBsAg Loss During Tenofovir-Containing Antiretroviral Therapy

	HBsAg Loss^a^	
	Participants Without (n = 29)	Participants With (n = 29)
Female sex at birth	6 (21)	6 (21)
Age, y	39 (36–46)	42 (38–46)
Calendar year of tenofovir start	2005 (2003–2007)	2005 (2003–2007)
Follow-up duration, y	11.1 (7.9–14.1)	12.3 (10.4–14.1)
European origin	14 (48)	22 (76)
Ethnicity		
White	16 (55)	22 (76)
Black	10 (34)	4 (14)
Asian	3 (10)	1 (3)
Other or unknown	0 (0)	2 (7)
Body mass index, kg/m^2^	22.7 (19.2–26.9)	22.9 (21.0–25.5)
Lamivudine pretreatment	24 (83)	24 (83)
Duration, y	6.3 (3.9–7.3)	6.2 (4.8–7.8)
CD4+ T-cell count <200 cells/µL	4 (14)	4 (14)
CD4/CD8 ratio	0.4 (0.3–0.6)	0.5 (0.3–0.7)
HIV viral load ≥50 copies/mL	15 (52)	13 (45)
Stage C^b^	10 (34)	8 (28)
HBV genotype		
A	11 (38)	8 (28)
A + G	0 (0)	3 (10)
C	1 (3)	0 (0)
D	2 (7)	4 (14)
Not available	15 (52)	14 (48)
HBV DNA, log_10_ IU/mL	4.0 (1.5–7.9)	3.0 (1.2–7.5)
Quantitative HBsAg		
log_10_ IU/mL	4.0 (3.5–4.2)	3.4 (2.1–4.5)
<1 log_10_ IU/mL	1 (3)	5 (17)
HBcrAg^c^		
log_10_ U/mL	5.6 (3.7–7.9)	6.9 (3.8–8.6)
≤3 log_10_ U/mL	4/28 (14)	4/26 (15)
HBV RNA^c^		
log_10_ copies/mL	3.3 (<1.0–5.7)	4.7 (<1.0–5.6)
<10 copies/mL	10/28 (36)	9/26 (35)
HBeAg positive	10/24 (42)	13/27 (48)
Alanine aminotransferase elevation	15 (52)	16 (55)

Data are presented as No. (%) or median (IQR).

Abbreviations: HBcrAg, hepatitis B core–related antigen; HBeAg, hepatitis B e antigen; HBsAg, hepatitis B surface antigen; HBV, hepatitis B virus.

^a^Defined as quantitative HBsAg <0.05 IU/mL.

^b^According to the clinical classification of HIV disease by the US Centers for Disease Control and Prevention.

^c^Measurements not available for 1 participant without HBsAg loss and 3 with HBsAg loss due to limited plasma sample volume.

### HBV RNA and HBcrAg Trajectories During Tenofovir-Containing ART

HBV RNA and HBcrAg levels at tenofovir start could be evaluated in 26 persons with HIV with HBsAg loss and 28 without loss who had sufficient plasma volume stored for these assessments. At tenofovir start, 65% of the participants with HBsAg loss and 64% of those without loss had detectable HBV RNA levels; HBcrAg was detectable in 85% of participants with HBsAg loss and 86% without loss (Table 1). A decline ≥ 1 log_10_ copies/mL in HBV RNA levels from baseline levels was observed in 88% of participants with HBsAg loss and 67% of those without loss after 2 years (*P* = .23). A decline ≥ 1 log_10_ U/mL in HBcrAg levels or newly negative HBcrAg after 2 years was observed in 73% of participants with HBsAg loss and in 36% without loss (*P* = .02).

**Figure 1.**
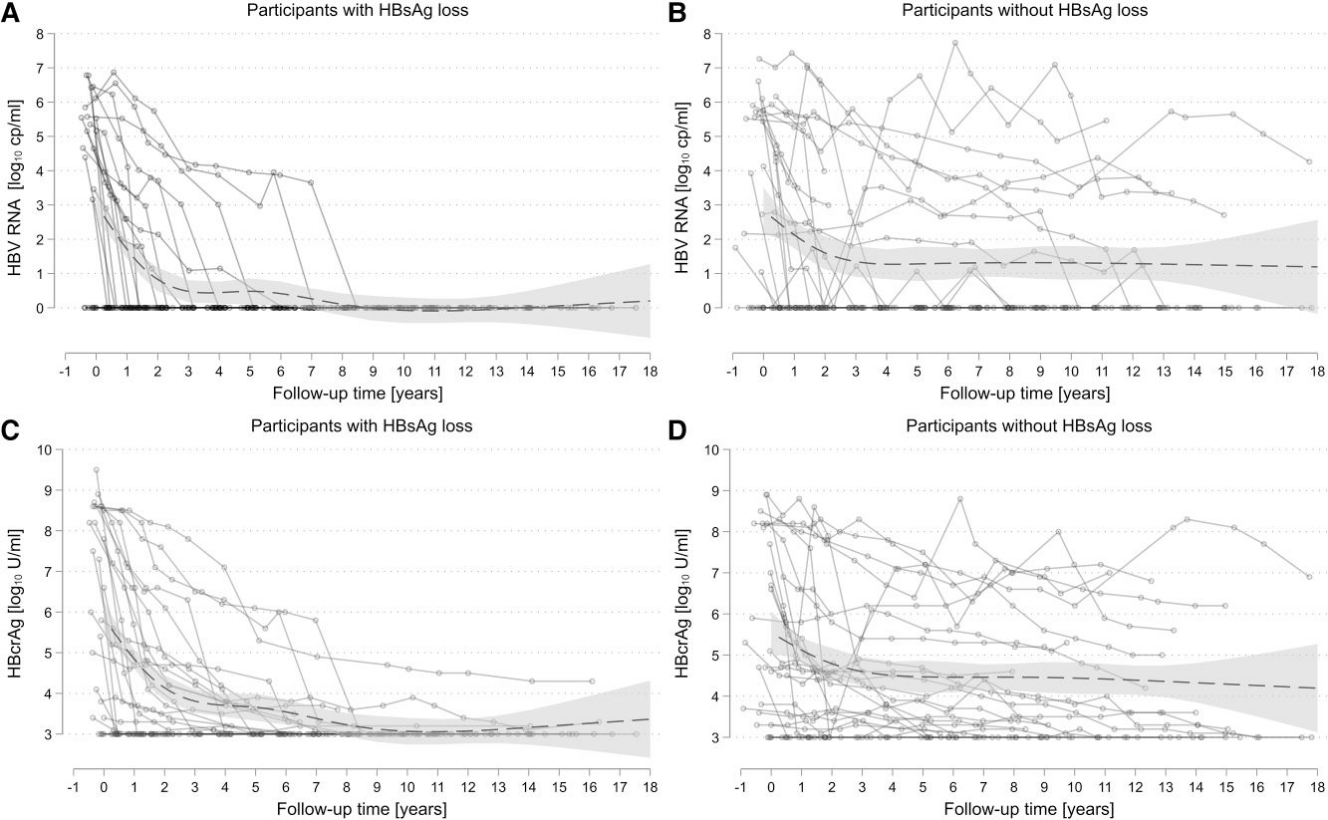
Circulating HBV RNA levels and HBcrAg levels in participants (*A*, *C*) with and (*B*, *D*) without HBsAg loss (defined as quantitative HBsAg <0.05 IU/mL) during tenofovir-containing antiretroviral therapy. HBV RNA and HBcrAg levels over time were modeled using linear regression while incorporating follow-up time as restricted cubic splines with 5 knots located at the 5th, 27.5th, 50th, 72.5th, and 95th percentiles. Follow-up time refers to time since start of tenofovir therapy. Circles with connecting solid lines, individual trajcetories; dashed line, modeled HBV RNA and HBcrAg levels; shaded area, 95% confidence intervals. cp, copies; HBcrAg, hepatitis B core–related antigen; HBsAg, hepatitis B surface antigen; HBV, hepatitis B virus.

The individual long-term trajectories of HBV RNA and HBcrAg levels are depicted in Figure 1. All participants with HBsAg loss reached undetectable HBV RNA levels during tenofovir-containing ART, as did 79% of those without loss (*P* = .02). All participants with HBsAg loss achieved undetectable HBV RNA before or at the time of HBsAg loss. In contrast, 19 (66%) participants had negative HBcrAg levels at the time of HBsAg loss, whereas 14% reached negative HBcrAg levels after HBsAg clearance and 21% had HBcrAg levels >3 log_10_ U/mL until the end of follow-up (Supplementary Figure 1). In comparison, 52% of the participants without HBsAg loss remained HBcrAg positive (*P* = .03). Combined HBV DNA suppression, undetectable HBV RNA, and negative HBcrAg during follow-up were achieved in 13 of 29 (45%) participants without HBsAg loss and was more likely in individuals who were HBeAg negative (9/14, 64%) than HBeAg positive (1/10, 10%; *P* = .01). Among participants with HBsAg loss, 23 of 29 (79%) reached combined HBV DNA suppression, undetectable HBV RNA, and negative HBcrAg, without significant differences between individuals who were HBeAg negative (12/14, 89%) and HBeAg positive (9/13, 69%; *P* = .38). Among the participants with HBsAg loss, 28 of 29 (97%) had sustained qHBsAg <0.05 IU/mL (ie, at least 2 consecutive samples with qHBsAg <0.05 IU/mL), whereas those without HBsAg loss all remained at qHBsAg ≥0.05 IU/mL throughout follow-up. One participant with HBsAg loss had a transient detectable level of qHBsAg coinciding with ART interruption. This participant was able to reach qHBsAg <0.05 IU/mL 4 years after this event, while HBcrAg remained negative and HBV RNA undetectable during this period. Supplementary Table 1 depicts the number of participants as well as the number of qHBsAg, HBV DNA, HBcrAg, and HBV RNA measurements at each follow-up time point.

### Cumulative Proportions With HBV DNA Suppression, Negative HBcrAg, and Undetectable HBV RNA Levels

**Figure 2.**
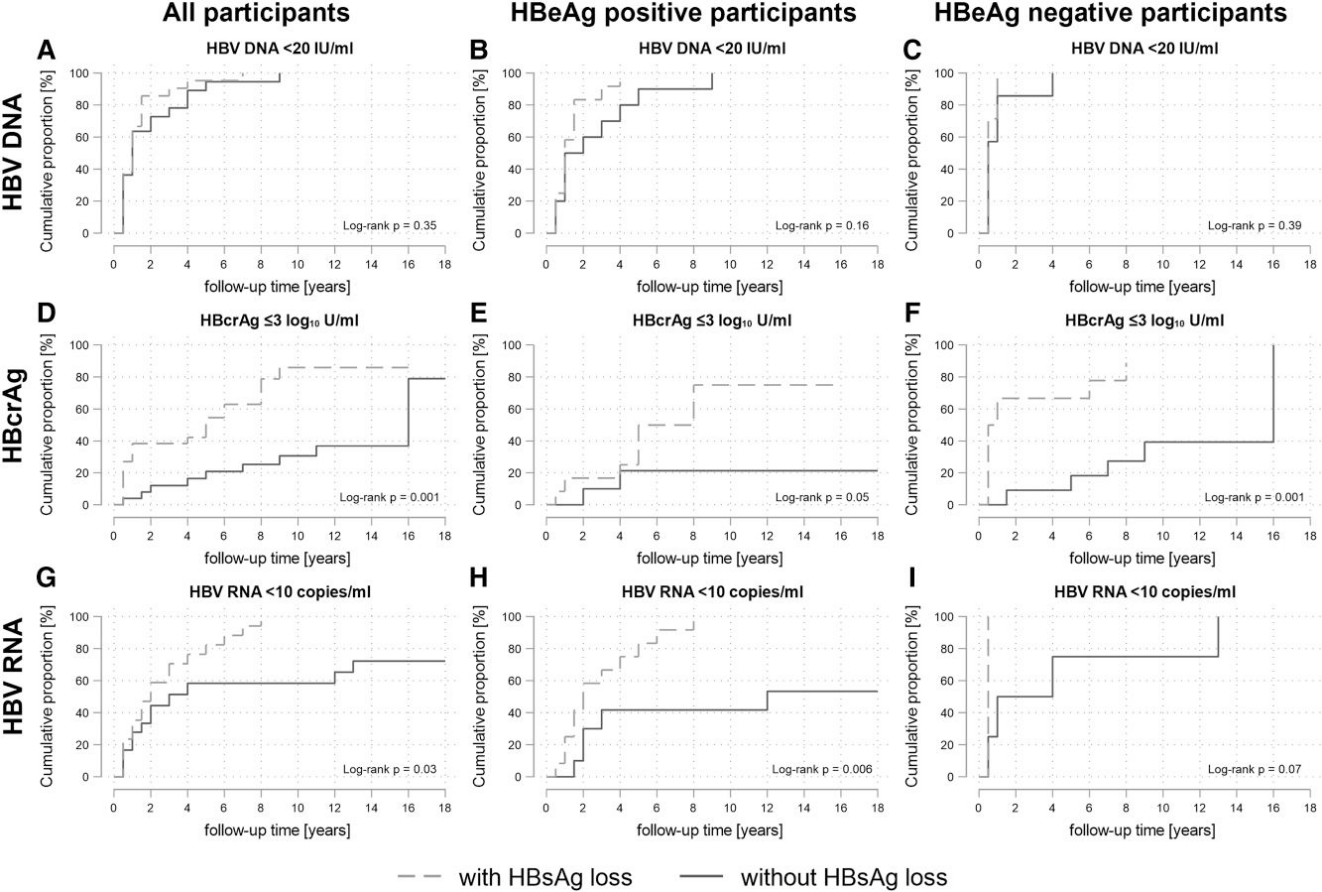
Kaplan-Meier curves for cumulative proportions with HBV DNA <20 IU/mL, HBcrAg level ≤3 log10 U/mL, and HBV RNA <10 copies/mL after starting tenofovir-containing antiretroviral therapy, stratified by participants with and without HBsAg loss. Cumulative proportions: *A*, *D*, *G*, all participants; *B*, *E*, *H*, participants who were HBeAg positive; *C*, *F*, *I*, participants who were HBeAg negative. Only participants with detectable levels at the start of tenofovir-containing antiretroviral therapy were included in the analysis; HBsAg loss was defined as quantitative HBsAg <0.05 IU/mL. HBcrAg, hepatitis B core–related antigen; HBsAg, hepatitis B surface antigen; HBV, hepatitis B virus.

Median time to HBV DNA suppression was similar in participants with and without HBsAg loss (12 months [95% CI, 6–18] vs 12 months [95% CI, 6–24], *P* = .35; Figure 2*A*). Among those with detectable levels at baseline, the Kaplan-Meier cumulative probabilities for HBcrAg ≤3 log_10_ U/mL (*P* = .001) and HBV RNA <10 copies/mL (*P* = .03) were significantly higher for participants with HBsAg loss as compared with those without loss (Figure 2*D* and 2*G*). The cumulative probabilities for HBcrAg ≤3 log_10_ U/mL for persons with and without HBsAg loss, stratified by HBeAg status, are shown in Figure 2*E* and 2*F*. The respective estimates for undetectable HBV RNA levels are presented in Figure 2*H* and 2*I*. Crude proportions of participants with HBV DNA suppression, negative HBcrAg, and undetectable HBV RNA levels at baseline and after 1, 2, 3, 5, and 10 years after tenofovir start are shown in Supplementary Figure 2.

### HBcrAg and HBV RNA as Predictive Markers for HBsAg Loss

In comparison with qHBsAg decline ≥1 log_10_ IU/mL, HBcrAg decline ≥1 log_10_ U/mL after 1 and 2 years had higher sensitivity but lower specificity in predicting HBsAg loss at 2 or 5 years. HBV RNA decline ≥1 log_10_ copies/mL after 1 and 2 years had 100% sensitivity in predicting HBsAg loss at 2 and 5 years but only 40.0% and 36.4% specificity (Table 2). A combination of HBcrAg or HBV RNA decline with qHBsAg decline did not improve sensitivity and showed a similar AUROC than qHBsAg decline alone. Supplementary Table 2 shows detailed reports of the performance of qHBsAg, HBcrAg, and HBV RNA as markers for HBsAg loss at 2 and 5 years based on time-dependent receiver operating characteristic curves. A combination of undetectable HBV RNA levels and HBcrAg decline ≥1 log_10_ U/mL after 1 year of tenofovir therapy revealed the highest AUROC (0.831) for the prediction of HBsAg loss after 2 years. For the prediction of HBsAg loss after 5 years, the highest AUROC values were identified with the combination of qHBsAg and HBcrAg decline ≥1 log_10_ (AUROC, 0.814) and in HBcrAg decline ≥1 log_10_ U/mL (AUROC, 0.810) 1 year after starting tenofovir.

**Table 2.** qHBsAg, HBcrAg, and HBV RNA Decline After 1 and 2 Years of Tenofovir-Containing ART as Predicting Markers for HBsAg Loss Within 2 and 5 Years Based on Time-Dependent AUROCs

	HBsAg Loss^a^ Within 2 y		
	Sensitivity, %	Specificity, %	AUROC
Decline ≥1 log_10_ after 1 y of tenofovir-containing ART^b^			
qHBsAg, IU/mL	70.0	88.1	0.791
HBcrAg, U/mL	87.5	64.7	0.761
HBV RNA, copies/mL	100.0	40.0	0.700
HBV RNA and HBcrAg	85.7	64.7	0.752
HBcrAg and qHBsAg	70.0	88.1	0.791
HBV RNA and qHBsAg	66.7	88.1	0.774

	HBsAg Loss^a^ Within 5 y		
Decline ≥1 log_10_ after 2 y of tenofovir-containing ART^b^			
qHBsAg, IU/mL	73.6	81.6	0.776
HBcrAg, U/mL	91.0	64.5	0.778
HBV RNA, copies/mL	100.0	36.4	0.682
HBV RNA and HBcrAg	89.0	67.7	0.784
HBcrAg and qHBsAg	69.4	89.2	0.793
HBV RNA and qHBsAg	66.9	88.6	0.777

Abbreviations: ART, antiretroviral therapy; AUROC, area under the receiver operating characteristic curve; HBcrAg, hepatitis B core–related antigen; HBsAg, hepatitis B surface antigen; HBV, hepatitis B virus; qHBsAg, quantitative hepatitis B surface antigen.

^a^Defined as qHBsAg <0.05 IU/mL.

^b^Details of this analysis are shown in Supplementary Table 2.

## DISCUSSION

In our study, we described the trajectories of HBcrAg and HBV RNA during a median follow-up of 12 years on tenofovir-containing ART in persons with and without HBsAg loss. Of the participants with HBsAg loss, all achieved undetectable circulating HBV RNA levels, and approximately 80% reached negative HBcrAg levels during follow-up. In persons without HBsAg loss, the probability of achieving undetectable HBV RNA and HBcrAg levels was significantly lower than in persons with HBsAg loss. A decline ≥1 log_10_ in HBV RNA or in HBcrAg levels after 2 years of tenofovir therapy had high sensitivity but low specificity for predicting HBsAg loss.

Undetectable HBV RNA levels preceded the first occurrence of undetectable qHBsAg levels in all participants with HBsAg loss. Similar findings were reported in a retrospective analysis including participants with HIV and HBV from 2 randomized controlled ART trials, where all but 1 participant with HBsAg loss had undetectable HBV RNA levels [21]. In line with these results, a study from Beijing in persons without HIV reported undetectable HBV RNA preceding HBsAg loss during nucleos(t)ide analogue therapy [26]. In contrast, a study from Taiwan found detectable HBV RNA levels at the time of HBsAg clearance in the majority of participants, but undetectable levels were achieved in all participants within the following 3 years [27]. Differences in the population characteristics, inclusion criteria, and technical characteristics of the assays used might explain these discrepancies. A recent study in 5 French centers reported undetectable circulating HBV RNA among all 27 persons with HBV 12 months after liver transplantation, whereas HBcrAg remained detectable in 30% of the transplant recipients [28]. Our results are in line with these findings, although the setting and population differ considerably between the studies. Similarly, a study from Hong Kong observed detectable HBcrAg levels in 12 of 55 persons without HIV who experienced spontaneous HBsAg loss [29]. Detectable HBcrAg despite undetectable HBV DNA and HBsAg may reflect ongoing low-level cccDNA transcriptional activity, while qHBsAg and HBV DNA may still be present in the serum below detection levels of the assays used in our study [28, 29]. Whether low-level transcriptional activity also has clinical implications with regard to the development of novel HBV drugs or for predicting the risk of hepatocellular carcinoma is currently uncertain.

A decline ≥ 1 log_10_ in HBV RNA or HBcrAg levels after 2 years of tenofovir-containing ART had higher sensitivity but low specificity for HBsAg loss than a decline in qHBsAg levels. A recent systematic review of 6 studies including 1257 persons reported the predictive value of HBcrAg levels for HBsAg loss with a median AUROC of 0.645 [30]. However, only 1 of these studies investigated the change in HBcrAg levels for predicting HBsAg loss during HBV therapy in persons without HIV with an AUROC of 0.521 [31]. A recent study including persons without HIV who were HBeAg positive and negative reported that a decline >2 log U/mL in HBcrAg after 4 weeks of antiviral therapy had a sensitivity and specificity for HBsAg loss of 75% and 62.5%, respectively [32]. In our study, baseline levels in HBcrAg and HBV RNA were not significantly different in both groups, consistent with previous reports on the limited use of baseline markers for predicting HBsAg loss [30]. As shown in Supplementary Table 2, a combination of negative HBV RNA and HBcrAg decline after 1 year of tenofovir therapy showed good performance in predicting HBsAg loss after 2 years and could serve as an alternative end point in the development of new HBV treatment strategies [33]. Adding qHBsAg decline to these 2 markers did not lead to further improvement in sensitivity and specificity. As these assays can be used on widely available diagnostic platforms, integrating these biomarkers into clinical care would be realistic in high-income countries. Yet, this would not be the case in low- and middle-income countries, where even HBV DNA measurements are often inaccessible due to high costs.

The majority of participants experienced a decline ≥ 1 log_10_ in HBcrAg and HBV RNA levels within 2 years of tenofovir therapy, even in the absence of HBsAg loss. Among participants without HBsAg loss, the combined suppression of HBV DNA, HBV RNA, and HBcrAg was more likely in participants who were HBeAg negative than HBeAg positive. Integrated DNA has been identified as the main source of HBsAg production in individuals who are HBeAg negative, which could explain the persistently detectable qHBsAg levels despite serologic evidence of cccDNA silencing as reflected by undetectable HBV RNA and HBcrAg levels [34, 35]. Previous studies found an association between (1) undetectable HBcrAg levels and HBV RNA levels and (2) favorable outcomes after cessation of nucleos(t)ide analogue therapy in persons without HIV who were HBeAg negative, but the generalizability of these findings to persons with HIV is limited as lifelong HBV-active therapy is recommended by current guidelines [3, 4, 36, 37].

Our study provides detailed insights into the kinetics of HBcrAg and HBV RNA in persons with HIV/HBV undergoing tenofovir-containing ART. We were able to compare the trajectories of both markers in individuals experiencing HBsAg loss with the trajectories in similar individuals not experiencing HBsAg loss using stringent inclusion and matching criteria. Despite the relatively small sample size due to the low frequency of HBsAg loss, our study design allowed us to involve one of the largest number of persons with HIV experiencing HBsAg loss during tenofovir therapy followed longitudinally to date. Several reports have highlighted the potential of circulating HBV RNA quantification to serve as a surrogate marker for intrahepatic cccDNA transcriptional activity and assessment of antiviral efficacy [17, 18, 38, 39]. The majority of currently available tests have a lower limit of quantification of around 1000 copies/mL, although in-house reverse transcription droplet digital polymerase chain reaction assays and the Abbott serum HBV RNA assay have lower limits of quantification of approximately 100 copies/mL [16, 40, 41]. In our study, we used a recently developed, highly sensitive investigational assay to quantify serum HBV RNA preferentially expressed from cccDNA with a lower limit of detection <5 copies/mL across a broad range of HBV genotypes, which ascertained robust results and potentially improves the diagnostic value of circulating HBV RNA in the prediction of HBsAg loss [19, 23]. However, currently available HBV RNA assays are not yet standardized, and detectable HBV RNA may not exclusively consist of pregenomic RNA. The applicability of HBcrAg is currently limited by the lower limit of sensitivity of the assay. To avoid false-positive results, we used a stringent cutoff of 1000 U/mL, as recommended by the manufacturer. An assay with a lower limit of sensitivity has recently been developed [42]. In addition, we were unable to correlate directly circulating HBV RNA and HBcrAg levels with intrahepatic cccDNA due to the absence of liver biopsies in this cohort. Moreover, HBeAg status at the start of tenofovir therapy was available in only 51 of 58 participants. Yet, the equal distribution of HBeAg status among participants with and without HBsAg loss should have limited any nondifferential bias with respect to this factor.

In conclusion, our findings indicate that in persons with HIV receiving tenofovir-containing ART, HBV RNA suppression precedes HBsAg loss. However, as HBV RNA clearance occurs in the majority of persons without HBsAg loss, its predictive value is limited. HBV RNA and HBcrAg declines during the first 2 years of tenofovir therapy have a high sensitivity for HBsAg loss, emphasizing the potential of these markers for the identification of individuals who will not experience HBsAg loss on tenofovir-containing ART. A combination of HBV RNA and HBcrAg levels over time could improve predictions of HBsAg loss in clinical trials of HBV drugs with novel modes of action.

## Supplementary Data

Supplementary materials are available at *The Journal of Infectious Diseases* online (http://jid.oxfordjournals.org/). Supplementary materials consist of data provided by the author that are published to benefit the reader. The posted materials are not copyedited. The contents of all supplementary data are the sole responsibility of the authors. Questions or messages regarding errors should be addressed to the author.

## Supplementary Material

jiae189_Supplementary_Data

## Notes

***Acknowledgments.*** We thank all patients, physicians, and nurses associated with the participating cohorts. The data are gathered by the 5 Swiss university hospitals, 2 cantonal hospitals, 15 affiliated hospitals, and 36 private physicians (listed in http://www.shcs.ch/180-health-care-providers).

***Members of the Swiss HIV Cohort Study.*** I. Abela, K. Aebi-Popp, A. Anagnostopoulos, M. Battegay, E. Bernasconi, D. L. Braun, H. C. Bucher, A. Calmy, M. Cavassini, A. Ciuffi, G. Dollenmaier, M. Egger, L. Elzi, J. Fehr, J. Fellay, H. Furrer, C. A. Fux, H. F. Günthard (president of the SHCS), A. Hachfeld, D. Haerry (deputy of Positive Council), B. Hasse, H. H. Hirsch, M. Hoffmann, I. Hösli, M. Huber, D. Jackson-Perry (patient representatives), C. R. Kahlert (chair of the Mother & Child Substudy), O. Keiser, T. Klimkait, R. D. Kouyos, H. Kovari, K. Kusejko (head of data center), N. Labhardt, K. Leuzinger, B. Martinez de Tejada, C. Marzolini, K. J. Metzner, N. Müller, J. Nemeth, D. Nicca, J. Notter, P. Paioni, G. Pantaleo, M. Perreau, A. Rauch (chair of the Scientific Board), L. Salazar-Vizcaya, P. Schmid, R. Speck, M. Stöckle (chair of the Clinical and Laboratory Committee), P. Tarr, A. Trkola, G. Wandeler, M. Weisser, and S. Yerly.

***Author contributions.*** L. B., A. B., G. W., A. R., M. L., and F. Z. designed the study. M.-L. P., B. T., F. S.-R., and C. S. performed the serologic and virologic analyses. L. B. and A. B. analyzed the data. L. B., G. W., and A. R. prepared the first draft, which was revised by all coauthors. C. B., H. F. G., A. C., M. C., H. H. H., P. S., E. B., G. W., and A. R. contributed to data acquisition. All authors contributed to the interpretation of the analyses.

***Data sharing.*** Data are not publicly available.

***Disclaimer.*** The funders had no role in study design, data collection and analysis, decision to publish, or preparation of the manuscript.

***Financial support*.** This work was supported by an investigator-initiated trial grant from Gilead Sciences (CO-SW-985-5602), by the NEAT-ID Foundation, by the Department of Teaching and Research, Inselspital, Bern University Hospital, and by the Liquid Biobank Inselspital Bern. This study has been financed within the framework of the SHCS, supported by the Swiss National Science Foundation (grant 201369), by SHCS projects 809 and 868, and by the SHCS research foundation. The SHCS biobank was supported by the Liquid Biobank Inselspital Bern and by the Department of Teaching and Research, Inselspital, Bern University Hospital. L. B. was supported by the Young Talents in Clinical Research program of the Swiss Academy of Medical Sciences and Gottfried and Julia Bangerter Rhyner Foundation (grant YTCR 13/19). G. W. was supported by a professorship from the Swiss National Science Foundation (PP00P3_211025). F. Z. and M. L. received public grants overseen by the French National Research Agency as part of the second Investissements d’Avenir Program (reference ANR-17-RHUS-0003) and by the European Union (grant EU H2020-847939-IP-cure-B).
