# Supplementary material for: Circulating HBV RNA and Hepatitis B Core–Related Antigen Trajectories in Persons With HIV/HBV Coinfection and Hepatitis B Surface Antigen Loss During Tenofovir Therapy
Source: J Infect Dis. 2024 Apr 16;230(4):e954–63. doi: 10.1093/infdis/jiae189 (PMC11481342; doi:10.1093/infdis/jiae189)
Supplement: jiae189_Supplementary_Data [file jiae189_supplementary_data.docx]

**SUPPLEMENTARY MATERIAL**

**Table of Contents**

[Supplementary Table 1: Number of participants followed and number of available qHBsAg, HBV DNA, HBcrAg and HBV RNA measurements per follow-up time point 2](#_Toc159220797)

[Supplementary Table 2. Predicting markers for HBsAg loss within two and five years using time-dependent receiver operating characteristic analysis 3](#_Toc159220798)

[Supplementary Figure 1. Difference in months between the time of HBsAg loss and the time of HBV RNA <10 copies/ml (A) or the time of HBcrAg ≤3 log_10_ U/ml (B) 4](#_Toc159220799)

[Supplementary Figure 2. Proportion of participants with HBV DNA <20 IU/ml, HBcrAg levels ≤3 log10 U/ml and HBV RNA <10 copies/ml at start of tenofovir therapy (baseline) and after 1, 2, 3, 5 and 10 years, stratified on participants with and without HBsAg loss 5](#_Toc159220800)

# **Supplementary Table 1: Number of participants followed and number of available qHBsAg, HBV DNA, HBcrAg and HBV RNA measurements per follow-up time point.**

|  | **Participants (N)** | **Follow-up time after tenofovir start (years)** | | | | | | | | | | | | | | | | | | | | |
| --- | --- | --- | --- | --- | --- | --- | --- | --- | --- | --- | --- | --- | --- | --- | --- | --- | --- | --- | --- | --- | --- | --- |
|  |  | **0** | **0.5** | **1** | **1.5** | **2** | **3** | **4** | **5** | **6** | **7** | **8** | **9** | **10** | **11** | **12** | **13** | **14** | **15** | **16** | **17** | **18** |
| with HBsAg loss | on follow-up | 29 | 29 | 29 | 29 | 29 | 29 | 29 | 28 | 28 | 25 | 25 | 22 | 22 | 20 | 15 | 14 | 11 | 7 | 5 | 2 | 1 |
|  | with qHBsAg measurement | 29 | 27 | 24 | 26 | 25 | 18 | 26 | 18 | 24 | 18 | 19 | 13 | 16 | 15 | 9 | 7 | 9 | 4 | 4 | 1 | 1 |
|  | with HBV DNA measurement | 29 | 27 | 24 | 26 | 26 | 18 | 26 | 18 | 23 | 17 | 18 | 13 | 14 | 13 | 9 | 7 | 8 | 4 | 4 | 1 | 1 |
|  | with HBcrAg measurement | 26 | 27 | 25 | 26 | 26 | 18 | 26 | 18 | 24 | 18 | 19 | 13 | 16 | 15 | 9 | 7 | 9 | 4 | 4 | 1 | 1 |
|  | with HBV RNA measurement | 26 | 27 | 25 | 26 | 26 | 18 | 26 | 18 | 24 | 18 | 19 | 13 | 16 | 15 | 9 | 7 | 9 | 4 | 4 | 1 | 1 |
| without HBsAg loss | on follow-up | 29 | 29 | 29 | 29 | 29 | 26 | 26 | 26 | 26 | 24 | 23 | 20 | 18 | 16 | 14 | 13 | 9 | 7 | 3 | 3 | 2 |
|  | with qHBsAg measurement | 29 | 22 | 26 | 23 | 26 | 23 | 24 | 25 | 24 | 23 | 22 | 19 | 15 | 13 | 11 | 12 | 9 | 7 | 3 | 1 | 2 |
|  | with HBV DNA measurement | 29 | 22 | 26 | 23 | 27 | 23 | 24 | 25 | 25 | 23 | 22 | 20 | 14 | 12 | 10 | 10 | 7 | 5 | 3 | 1 | 2 |
|  | with HBcrAg measurement | 28 | 22 | 26 | 23 | 27 | 23 | 24 | 25 | 25 | 23 | 22 | 20 | 15 | 14 | 11 | 12 | 9 | 7 | 3 | 1 | 2 |
|  | with HBV RNA measurement | 28 | 22 | 26 | 23 | 27 | 23 | 24 | 25 | 25 | 23 | 22 | 20 | 15 | 14 | 11 | 12 | 9 | 7 | 3 | 1 | 2 |

^Abbreviations: DNA, deoxyribonucleic acid; HBcrAg, hepatitis B core-related antigen; HBsAg, hepatitis B surface antigen; HBV, hepatitis B virus; qHBsAg, quantitative hepatitis B surface antigen; RNA, ribonucleic acid.^

# **Supplementary Table 2. Predicting markers for HBsAg loss* within two and five years using time-dependent receiver operating characteristic analysis.**

|  | HBsAg loss within 24 months† | | | | | HBsAg loss within 60 months‡ | | | | |
| --- | --- | --- | --- | --- | --- | --- | --- | --- | --- | --- |
|  | HBsAg loss (n) | No loss (n) | Sensitivity (%) | Specificity (%) | AUC | HBsAg loss (n) | No loss (n) | Sensitivity (%) | Specificity (%) | AUC |
| Analysis of single biomarkers |  |  |  |  |  |  |  |  |  |  |
| qHBsAg |  |  |  |  |  |  |  |  |  |  |
| qHBsAg decline ≥1 log_10_ IU/ml after 1 yr | 10 | 42 | 70.0 | 88.1 | 0.791 | 15 | 38 | 59.4 | 92.1 | 0.758 |
| qHBsAg decline ≥1 log_10_ IU/ml after 2 yr | - | - | - | - | - | 15 | 38 | 73.6 | 81.6 | 0.776 |
| HBcrAg |  |  |  |  |  |  |  |  |  |  |
| HBcrAg decline ≥1 log_10_ U/ml after 1 yr§¶ | 8 | 34 | 87.5 | 64.7 | 0.761 | 11 | 31 | 91.0 | 71.0 | 0.810 |
| HBcrAg decline ≥1 log_10_ U/ml after 2 yr¶ | - | - | - | - | - | 11 | 31 | 91.0 | 64.5 | 0.778 |
| HBcrAg ≤3 log_10_ U/ml after 1 yr | 10 | 42 | 50.0 | 76.2 | 0.631 | 15 | 38 | 53.3 | 79.0 | 0.661 |
| HBcrAg ≤3 log_10_ U/ml after 2 yr | - | - | - | - | - | 15 | 38 | 53.3 | 76.3 | 0.648 |
| HBV RNA |  |  |  |  |  |  |  |  |  |  |
| HBV RNA decline ≥1 log_10_ copies/ml after 1 yr¶# | 6 | 25 | 100.0 | 40.0 | 0.700 | 8 | 22 | 100.0 | 45.5 | 0.722 |
| HBV RNA decline ≥1 log_10_ copies/ml after 2 yr¶ | - | - | - | - | - | 8 | 22 | 100.0 | 36.4 | 0.682 |
| HBV RNA <10 cp/ml after 1 yr | 10 | 42 | 90.0 | 50.0 | 0.700 | 15 | 38 | 79.7 | 50.0 | 0.649 |
| HBV RNA <10 cp/ml after 2 yr | - | - | - | - | - | 15 | 38 | 92.9 | 34.2 | 0.636 |
| Analysis of combined biomarkers‖ |  |  |  |  |  |  |  |  |  |  |
| HBV RNA and HBcrAg |  |  |  |  |  |  |  |  |  |  |
| HBV RNA <10 cp/ml and HBcrAg decline ≥1 log_10_ U/ml after 1 yr§¶ | 8 | 34 | 75.0 | 91.2 | 0.831 | 11 | 31 | 63.1 | 93.6 | 0.783 |
| HBV RNA <10 cp/ml and HBcrAg decline ≥1 log_10_ U/ml after 2 yr¶ | - | - | - | - | - | 11 | 31 | 81.2 | 77.4 | 0.793 |
| HBV RNA [cp/ml] and HBcrAg [U/ml] decline ≥1 log_10_ after 1 yr¶# | 7 | 34 | 85.7 | 64.7 | 0.752 | 9 | 31 | 89.0 | 71.0 | 0.800 |
| HBV RNA [cp/ml] and HBcrAg [U/ml] decline ≥1 log_10_ after 2 yr¶ | - | - | - | - | - | 9 | 31 | 89.0 | 67.7 | 0.784 |
| qHBsAg and HBcrAg |  |  |  |  |  |  |  |  |  |  |
| qHBsAg [IU/ml] and HBcrAg [U/ml] decline ≥1 log_10_ after 1 yr§¶ | 10 | 42 | 70.0 | 88.1 | 0.791 | 14 | 38 | 56.6 | 91.1 | 0.743 |
| qHBsAg [IU/ml] and HBcrAg [U/ml] decline ≥1 log_10_ after 2 yr¶ | - | - | - | - | - | 13 | 37 | 69.4 | 89.2 | 0.793 |
| qHBsAg [IU/ml] or HBcrAg [U/ml] decline ≥1 log_10_ after 1 yr¶ | 8 | 34 | 87.5 | 64.7 | 0.761 | 12 | 31 | 91.7 | 71.0 | 0.814 |
| qHBsAg [IU/ml] or HBcrAg [U/ml] decline ≥1 log_10_ after 2 yr¶ | - | - | - | - | - | 13 | 32 | 92.4 | 56.3 | 0.743 |
| HBcrAg ≤3 log_10_ U/ml or qHBsAg decline ≥1 log_10_ IU/ml after 1 yr | 10 | 42 | 90.0 | 66.7 | 0.783 | 15 | 38 | 86.3 | 71.1 | 0.787 |
| HBcrAg ≤3 log_10_ U/ml or qHBsAg decline ≥1 log_10_ IU/ml after 2 yr | - | - | - | - | - | 15 | 38 | 93.4 | 60.5 | 0.770 |
| HBV RNA and qHBsAg |  |  |  |  |  |  |  |  |  |  |
| HBV RNA <10 cp/ml and qHBsAg decline ≥1 log_10_ IU/ml after 1 yr | 10 | 42 | 60.0 | 95.2 | 0.776 | 15 | 38 | 46.2 | 97.4 | 0.718 |
| HBV RNA <10 cp/ml and qHBsAg decline ≥1 log_10_ IU/ml after 2 yr | - | - | - | - | - | 15 | 38 | 66.5 | 86.8 | 0.767 |
| HBV RNA [cp/ml] and qHBsAg [IU/ml] decline ≥1 log_10_ after 1 yr§¶ | 9 | 42 | 66.7 | 88.1 | 0.774 | 13 | 38 | 53.3 | 92.1 | 0.727 |
| HBV RNA [cp/ml] and qHBsAg [IU/ml] decline ≥1 log_10_ after 2 yr¶ | - | - | - | - | - | 12 | 35 | 66.9 | 88.6 | 0.777 |
| HBV RNA <10 cp/ml or qHBsAg decline ≥1 log_10_ IU/ml after 1 yr | 10 | 42 | 60.0 | 95.2 | 0.714 | 15 | 38 | 46.2 | 97.4 | 0.688 |
| HBV RNA <10 cp/ml or qHBsAg decline ≥1 log_10_ IU/ml after 2 yr | - | - | - | - | - | 15 | 38 | 66.5 | 86.8 | 0.645 |
| qHBsAg [IU/ml], HBcrAg [U/ml] and HBV RNA [cp/ml] decline >1 log_10_ after 1yr§¶ | 9 | 42 | 66.7 | 88.1 | 0.774 | 13 | 38 | 53.3 | 92.1 | 0.727 |
| qHBsAg [IU/ml], HBcrAg [U/ml] and HBV RNA [cp/ml] decline >1 log_10_ after 2 yr¶ | - | - | - | - | - | 12 | 37 | 66.9 | 89.2 | 0.780 |
| qHBsAg [IU/ml] and HBcrAg [U/ml] decline >1 log_10_ and HBV RNA <10cp/ml after 1 yr§¶ | 10 | 42 | 60.0 | 95.2 | 0.776 | 13 | 37 | 42.4 | 97.4 | 0.699 |
| qHBsAg [IU/ml] and HBcrAg [U/ml] decline >1 log_10_ and HBV RNA <10cp/ml after 2 yr¶ | - | - | - | - | - | 13 | 37 | 61.2 | 94.6 | 0.779 |

^* defined as qHBsAg <0.05 IU/ml, † six participants censored, unless otherwise specified, ‡ five participants censored, unless otherwise specified, § five participants censored at 24 months, ¶ N < 58 due to participants with missing information to calculate criteria (HBV RNA and/or HBcrAg below detection limit at baseline), # four participants censored at 24 months, ‖ only combinations with a sensitivity and specificity ≥60% are shown. Abbreviations: AUC, area under the curve; HBcrAg, hepatitis B core-related antigen; HBsAg, hepatitis B surface antigen; HBV, hepatitis B virus; IU/ml, international units per millilitre; qHBsAg, quantitative hepatitis B surface antigen; U/ml units per millilitre; yr, years.^

^^

# **Supplementary Figure 1. Difference in months between the time of HBsAg loss and the time of HBV RNA <10 copies/ml (A) or the time of HBcrAg ≤3 log_10_ U/ml (B).**

Every line represents one participant; lines without a bar reflect participants without a difference between the time of HBsAg loss and the time of HBV RNA <10 copies/ml or HBcrAg. ≤3 log_10_ U/ml.
* participant never achieved HBcrAg ≤3 log_10_ but experienced HBsAg loss, defined as qHBsAg <0.05 IU/ml. Abbreviations: HBcrAg, hepatitis B core-related antigen; HBV, hepatitis B virus; qHBsAg, quantitative hepatitis B surface antigen; U/ml, units per milliliter.

^
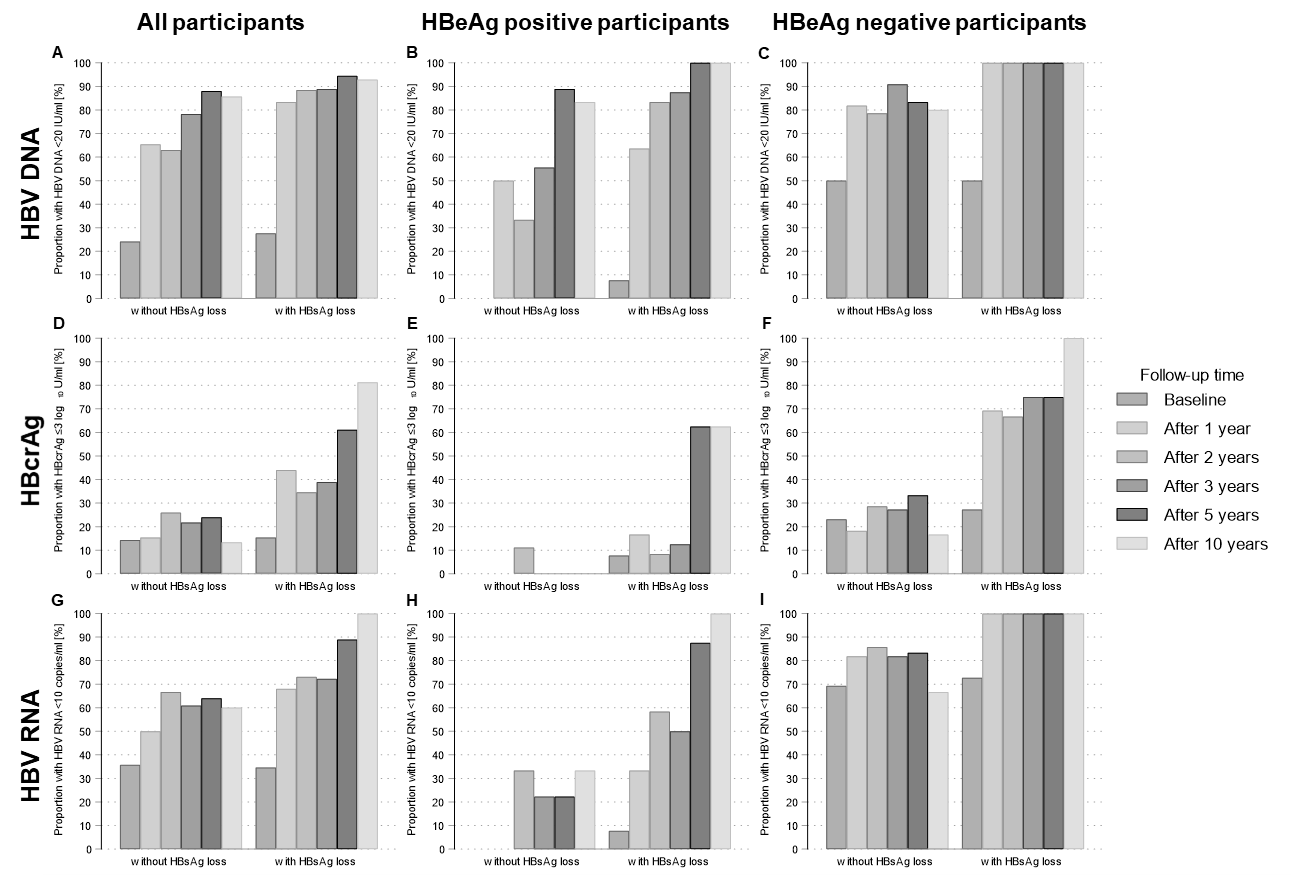
^

**Supplementary Figure 2. Proportion of participants with HBV DNA <20 IU/ml, HBcrAg levels ≤3 log10 U/ml and HBV RNA <10 copies/ml at start of tenofovir therapy (baseline) and after 1, 2, 3, 5 and 10 years, stratified on participants with and without HBsAg loss*.**
Panels A, D and G describe proportions for all participans; panels B, E and H describe proportions restricted to HBeAg-positive participants; and panels C, F and I describe proportions restricted to HBeAg-negative participants.
* defined as qHBsAg <0.05 IU/ml. Abbreviations: HBcrAg, hepatitis B core-related antigen; HBeAg, hepatitis B e antigen; HBV, hepatitis B virus; qHBsAg, quantitative hepatitis B surface antigen; U/ml, units per milliliter.
